# Genome Sequencing and Analysis of Catopsilia pomona nucleopolyhedrovirus: A Distinct Species in Group I *Alphabaculovirus*

**DOI:** 10.1371/journal.pone.0155134

**Published:** 2016-05-11

**Authors:** Jun Wang, Zheng Zhu, Lei Zhang, Dianhai Hou, Manli Wang, Basil Arif, Zheng Kou, Hualin Wang, Fei Deng, Zhihong Hu

**Affiliations:** 1 State Key Laboratory of Virology and China Center for Virus Culture Collection, Wuhan Institute of Virology, Chinese Academy of Sciences, Wuhan, China; 2 Canadian Forest Service, Great Lakes Forestry Centre, Sault Ste Marie, Ontario, Canada; Wuhan Bioengineering Institute, CHINA

## Abstract

The genome sequence of Catopsilia pomona nucleopolyhedrovirus (CapoNPV) was determined by the Roche 454 sequencing system. The genome consisted of 128,058 bp and had an overall G+C content of 40%. There were 130 hypothetical open reading frames (ORFs) potentially encoding proteins of more than 50 amino acids and covering 92% of the genome. Among all the hypothetical ORFs, 37 baculovirus core genes, 23 lepidopteran baculovirus conserved genes and 10 genes conserved in Group I alphabaculoviruses were identified. In addition, the genome included regions of 8 typical baculoviral homologous repeat sequences (*hr*s). Phylogenic analysis showed that CapoNPV was in a distinct branch of clade “a” in Group I alphabaculoviruses. Gene parity plot analysis and overall similarity of ORFs indicated that CapoNPV is more closely related to the Group I alphabaculoviruses than to other baculoviruses. Interesting, CapoNPV lacks the genes encoding the fibroblast growth factor (*fgf*) and *ac30*, which are conserved in most lepidopteran and Group I baculoviruses, respectively. Sequence analysis of the F-like protein of CapoNPV showed that some amino acids were inserted into the fusion peptide region and the pre-transmembrane region of the protein. All these unique features imply that CapoNPV represents a member of a new baculovirus species.

## Introduction

Members of the family *Baculoviridae* are rod-shaped, insect-specific viruses with double-stranded large circular DNA genomes of 80–180 kb [1, 2]. Lepidopteran baculoviruses synthesize two progeny phenotypes, the budded virus (BV) and occlusion-derived virus (ODV). Virus particles of the latter phenotype are embedded into occlusion bodies (OBs) [3], which offer some protection against environmental inactivating conditions such as UV light, heat and desiccation.

*Baculoviridae* contains four genera: *Alphabaculovirus* [nucleopolyhedroviruses (NPVs) of lepidopteran insects], *Betabaculovirus* [granuloviruses (GVs) of Lepidoptera], *Gammabaculovirus* (NPVs of Hymenoptera) and *Deltabaculovirus* (NPVs of Diptera) [4, 5]. The alphabaculoviruses can be further divided into Group I and Group II, based on phylogenetic analysis and their membrane fusion proteins. Group I viruses use GP64 as the fusion protein while Group II viruses use F-protein instead [6–8]. Phylogeny analysis suggested that Group I fall into two clades, “a” and “b” [9]. Despite the diversity in gene content of baculovirus genomes, 37 have been identified as core genes present in all sequenced baculoviral genomes and play very important roles in the viral replication cycle [10]. In addition, there are 23 genes conserved in all sequenced lepidopteran baculoviruses (NPVs and GVs) and 11 are specific to Group I [10–13].

*Catopsilia pomona* (Lepidoptera: Pieridae) is distributed in Asia and Australia. In Mainland China, it is present mainly in the provinces of Hainan, Guangdong, Guangxi, Yunnan, and Fujian. It is harmful to Kassod tree, Wing-podded Senna, golden shower, pockwood and other tropical plants[14]. Larvae feed on young leaves and during outbreaks, the trees are stripped of foliage totally. In Hainan Province, the insect has 13–14 generations a year, causing damage all year round [15]. CapoNPV was isolated from dead *Catopsilia pomona* larvae in Hainan in 1990 [15].

So far, 78 baculoviruses have been fully sequenced, including 19 Group I alphabaculoviruses, 35 Group II alphabaculoviruses, 20 betabaculoviruses, 3 gammabaculoviruses and 1 deltabaculovirus (http://www.ncbi.nlm.nih.gov/genomes/GenomesGroup.cgi?taxid=10442, and S1 Table). In this study, the complete genomic sequence of CapoNPV was determined and analyzed. Phylogenetic analysis suggested that this virus is a distinct species in Group I *Alphabaculovirus*.

## Results and Discussion

### Sequencing and genome characteristics

**Fig 1:**
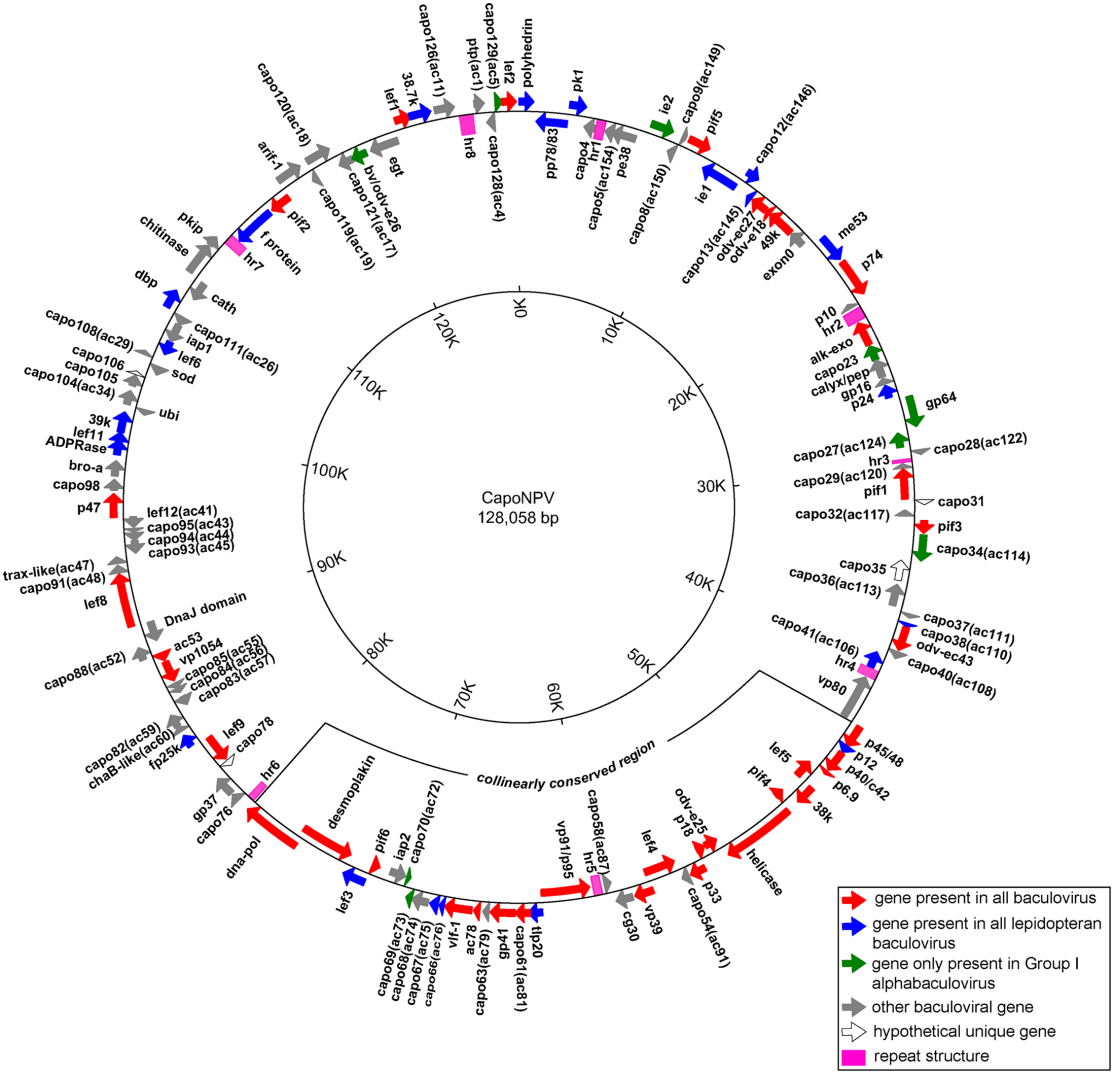
The circular map of CapoNPV. ORFs and direction of transcription are indicated by arrows. The colors represent gene types: red for core genes, blue for lepidopteran conserved genes, green for Group I specific genes, grey for other baculoviral genes. Open arrows represent hypothetical unique genes of CapoNPV. *Hr*s are represented by pink square boxes. The collinear region conserved in lepidopteran baculoviruses is also indicated.

The complete nucleotide sequence of CapoNPV genomic DNA was determined using 454 pyrosequencing method. The sequences were assembled using the Roche GS De Novo Assembler version 2.7. The genome was covered 350 times by 123,698 reads. It consists of 128,058 bp in length and contains 130 predicted ORFs with a G+C content of 40% (S2 Table). The adenine residue of the translation initiation codon of *polyhedrin* with a forward orientation was designated as the zero point on the circular genome map. Sixty-nine ORFs were in a clockwise direction and 61 in a counterclockwise direction with respect to the transcriptional orientation of *polyhedrin*. The 37 core genes (red), 23 lepidopteran baculovirus conserved genes (blue) and 10 Group I specific genes (green) are illustrated on the genome map (Fig 1). Another 56 baculoviral genes and 4 hypothetical CapoNPV unique genes are shown in grey and open arrows, respectively (Fig 1).

### Phylogenetic analysis of CapoNPV

**Fig 2:**
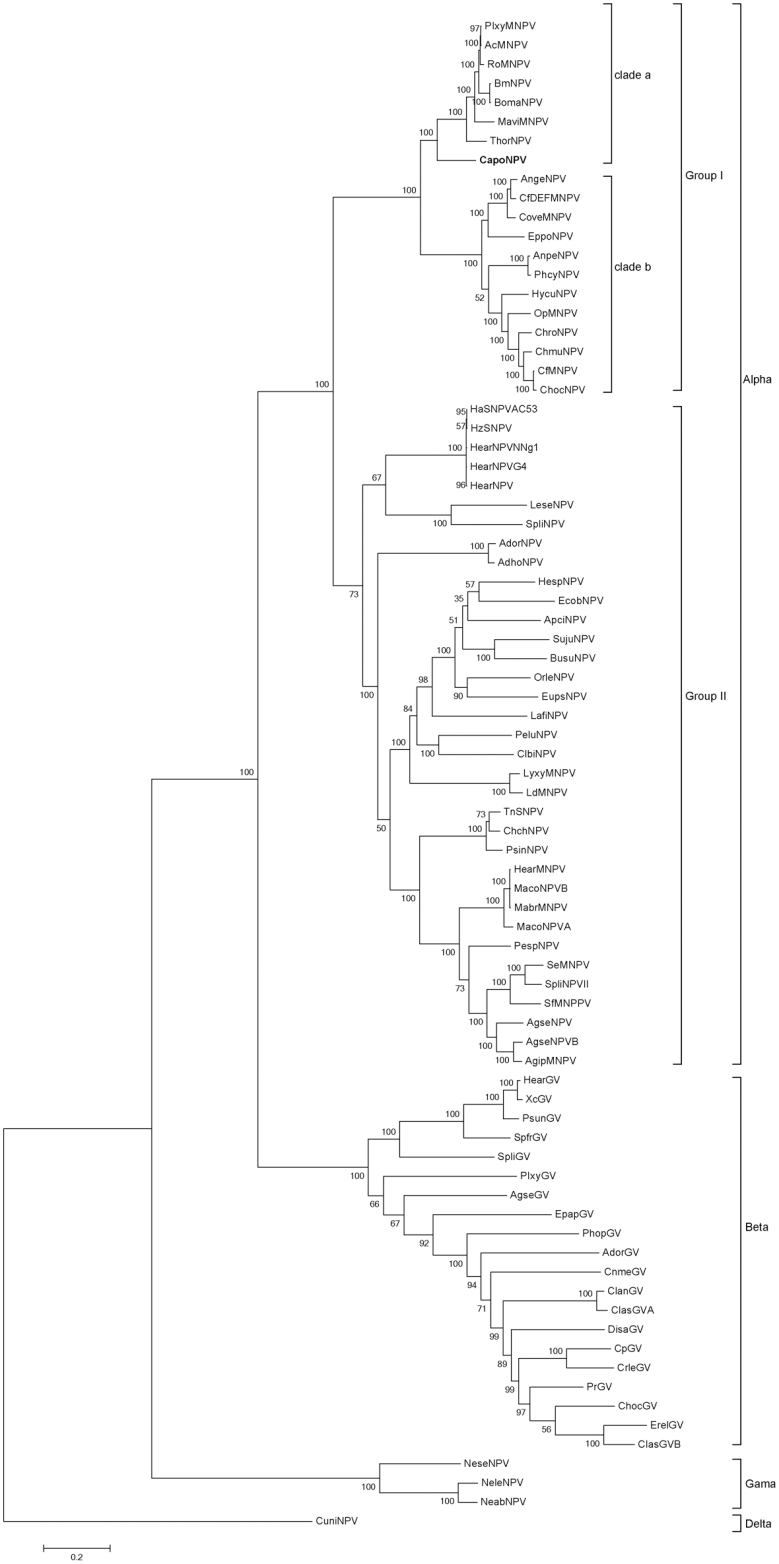
Phylogenetic tree. Phylogenetic analysis was performed using amino acid sequence alignments of the combined 37 core genes from 79 sequenced baculoviruses with Maximum Likelihood method. It is tested by Bootstrap method with a value of 1000. Numbers at nodes indicate bootstrap scores, only the value >50% are shown. CapoNPV is highlighted in bold.

A phylogenetic tree built with linked 37 core genes from 79 sequenced baculoviruses (S1 Table) classified CapoNPV into clade “a” of Group I (Fig 2). It is located on a distinct branch in clade “a”alphabaculoviruses, which is consistent with a previous phylogenetic analysis based on *polyhedrin/granulin*, *lef-8* and *lef-9* [9]. CapoNPV appeared to have diverged shortly after the separation of clades “a” and “b” and may be closer to an ancestral virus than most species in the two clades. This situation is similar to a newly sequenced Cyclophragma undans nucleopolyhedrovirus (CyunNPV) (data not shown).

### Comparison of CapoNPV ORFs to other baculoviruses

CapoNPV genes were compared to homologues in 7 other well characterized baculoviruses; Autographa californica MNPV(AcMNPV, belonging to Group I, clade “a”), Orgyia pseudotsugata MNPV, (OpMNPV, Group I, clade “b”), Helicoverpa armigera NPV(HearNPV, Group II), Spodoptera exigua MNPV (SeMNPV, Group II), Cydia pomonella GV (CpGV, a betabaculovirus), Neodiprion lecontei NPV (NeleNPV, a gammabaculovirus) and Culex nigripalpus NPV (CuniNPV, a deltabaculovirus) (S2 Table). Most of the CapoNPV genes shared nt identity lower than 63% with the alphabaculoviruses and lower than 35% with that of beta-, gamma- and deltabaculoviruses (S2 Table).

**Fig 3:**
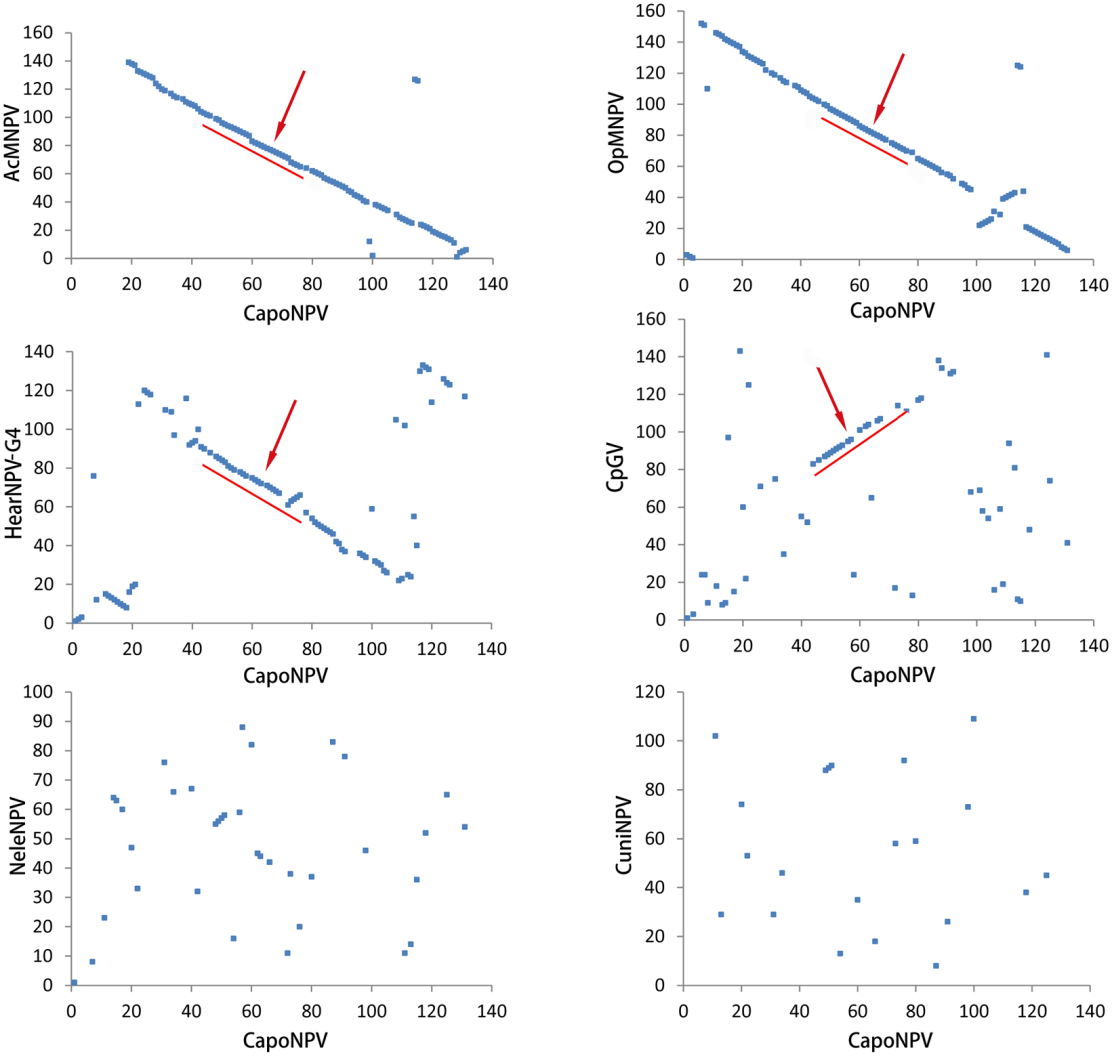
Gene parity plot analysis. Gene parity plots of CapoNPV against representative baculoviruses: AcMNPV (Group I clade “a”), OpMNPV (Group I clade “b”); HearNPV (Group II); CpGV (a betabaculovirus), NeleNPV (a gammabaculovirus) and CuniNPV (a deltabaculovirus). CapoNPV ORFs are on the X axis. The red line and arrow point to the collinearly conserved region.

Gene order of CapoNPV was compared to the above baculovirus genomes using gene parity plots [16]. Although CapoNPV is a distinct species in Group I, its gene order is substantially collinear with representatives of Group I alphabaculoviruses and partially collinear with those from Group II alphabaculoviruses. However, its gene arrangement was significantly different from that of gamma- and deltabaculoviruses (Fig 3). A collinearly conserved region of lepidopteran baculoviruses was also found in CapoNPV between *capo43* to *capo75* (Fig 1). It contains 20 core genes and five additional lepidopteran baculovirus conserved genes, and also includes two Group I specific genes, *ac73* (*capo69*) and *ac72* (*capo70*), and six other genes *ac91* (*capo58*), *cg30* (*capo57*), *ac87* (*capo58*), *ac79* (*capo63*), *ac74* (*capo68*) *and iap-2* (*capo71*) (Fig 1).

### Regions of homologous repeated sequences

**Fig 4:**
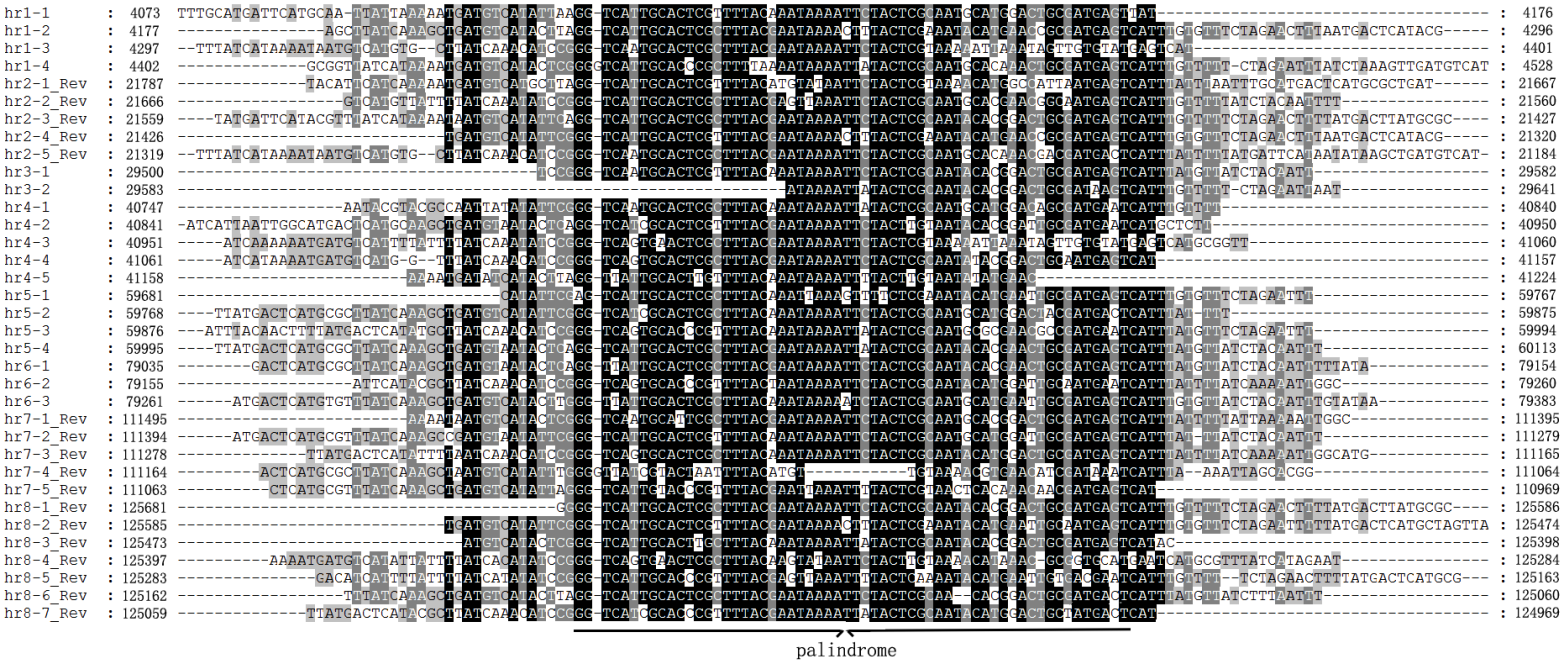
Sequence alignment of CapoNPV *hr*s. Black background shows greater than 80% identity among compared regions, dark gray and light gray shows greater than 50% and 30% identity, respectively. Palindromes are indicated below the alignments.

Homologous repeated sequences (*hr*s) of baculoviruses consist of a number of repeated sequences with an imperfect palindrome, interspersed at different locations in a genome. *Hrs* are highly variable, and although they are closely similar within the same genome, they may show very limited homology among different viruses. Sixty-four of the 79 completely sequenced baculoviral genomes contain 2–17 *hr*s (S1 Table). Previous studies suggested that *hrs* may act as origins of DNA replication [17, 18]. However, deletion of individual *hrs* from the AcMNPV genome does not appear to affect genome replication [19]. The *hr*s also acted as enhancers of gene expression and appeared to up-regulate the expression of the AcMNPV immediate early gene-1 (*ie-1*) [20–22]. The locations and the sequences of the 8 CapoNPV *hr*s are summarized in Figs 1 and 4, respectively.

### Gene content of CapoNPV

CapoNPV contains 12 replication associated genes, 12 transcription associated genes, 8 genes essential for oral infection, 34 structure related genes and 15 auxiliary genes (Table 1). The rest are 45 of unknown function including 4 hypothetical unique genes of CapoNPV.

**Table 1:** Gene contents of CapoNPV*.

Gene types	Core genes	Lepidoptera conserved genes	Other baculoviral genes	Missing genes
Replication	*alk-exo*(*capo21*), *dna polymerase*(*capo75*), *helicase*(*capo50*), *lef1*(*capo124*), *lef2*(*capo130*)	*dbp*(*capo112*), *ie-1*(*capo11*), *lef3*(*capo73*), *lef11*(*capo101*), *me53*(*capo18*)	*ac79*(*capo63*), *lef12*(*capo96*)	*lef7*
Transcription	*lef4*(*capo55*), *lef5*(*capo47*), *lef8*(*capo90*), *lef9*(*capo79*), *p47*(*capo97*), *vlf-1*(*capo65*)	*39k*(*capo102*), *lef6*(*capo109*), *pk-1*(*capo3*)	*exon0*(*capo17*), *ie-2*(*capo7*), *pe38*(*capo6*)	
Structure	*38k*(*capo48*), *49k*(*capo16*), *ac53*(*capo87*), *ac78*(*capo64*), *ac81*(*capo61*), *desmoplakin*(*capo74*), *gp41*(*capo62*), *odv-e18*(*capo15*), *odv-e25*(*capo51*), *odv-ec27*(*capo14*), *odv-ec43*(*capo39*), *p18*(*capo52*), *p33*(*capo53*), *p40*(*capo45*), *p48/p45*(*capo43*), *p6*.*9*(*capo46*), *vp1054*(*capo86*), *vp39*(*capo56*), *vp91/p95*(*capo59*)	*F*(*capo116*), *fp/25k*(*capo80*), *p12*(*capo44*), *p24*(*capo25*), *polyhedrin*(*capo1*), *tlp-20*(*capo60*)	*calyx/pep*(*capo23*), *cg30* (*capo57*), *gp16*(*capo24*), *gp64*(*capo26*), *odv-e26*(*capo122*), *p10*(*capo20*), *p78/83*(*capo2*), *pkip*(*capo115*), *vp80*(*capo42*)	*odv-e66*
Oral infection	*p74*(*capo19*), *pif1*(*capo30*), *pif2*(*capo117*), *pif3*(*capo33*), *pif4*(*capo49*), *pif5*(*capo10*), *pif6*(*capo72*)		*sf58*(*capo40*)	
Auxiliary		*38*.*7k*(*capo125*), *ADPRase*(*capo100*), *ubiquitin*(*capo103*)	*arif*(*capo118*), *bro-a*(*capo99*), *chitinase*(*capo114*), *djbp*(*capo89*), *egt*(*capo123*), *gp37*(*capo77*), *ptp*(*capo127*), *iap-1*(*capo110*), *iap-2*(*capo71*), *sod*(*capo107*), *trax-like*(*capo92*), *cath*(*capo113*)	*ac105*, *ac30*, *ctl*, *fgf*, *gta*, *MTase*, *p26*, *p94*
Unknown		*ac106*(*capo41*), *ac108*(*capo40*), *ac145*(*capo13*), *ac146*(*capo12*), *ac75*(*capo67*), *ac76*(*capo66*)	*ac11(capo126)*, *ac110(capo38)*, *ac111(capo37)*, *ac113(capo36)*, *ac114(capo34)*, *ac117(capo32)*, *ac12(capo98)*, *ac120(capo29)*, *ac122(capo28)*, *ac124(capo27)*, *ac132(capo22)*, *ac149(capo9)*, *ac150(capo8)*, *ac154(capo5)*, *ac17(capo121)*, *ac18(capo120)*, *ac19(capo119)*, *ac26(capo111)*, *ac29(capo108)*, *ac34(capo104)*, *ac4(capo128)*, *ac43(capo95)*, *ac44(capo94)*, *ac45(capo93)*, *ac48(capo91)*, *ac5(capo129)*, *ac52(capo88)*, *ac55(capo85)*, *ac56(capo84)*, *ac57(capo83)*, *ac59(capo82)*, *ac72(capo70)*, *ac73(capo69)*, *ac74(capo68)*, *ac87(capo58)*, *ac91(capo54)*, *capo105*, *capo4*, *capo76*	

* The CapoNPV hypothetical unique genes (*capo31*, *capo35*, *capo78* and *capo106*) are not included.

#### CapoNPV lacks fibroblast growth factor gene (*fgf*)

FGF plays an important role in developmental processes affecting cell growth, differentiation, and motility and is one of the conserved proteins in vertebrates and invertebrates [23]. Lepidopteran baculoviruses also encode *fgf*, and it was previously found conserved in all the lepidopteran baculoviruses [9] except in Maruca vitrata nucleopolyhedrovirus (MaviNPV) [12]. Although deletion of *fgf* from AcMNPV had no effect on replication in tissue culture cells, bioassays showed that time of death in larvae was delayed [24]. It has been suggested that FGF may play a role in dissemination of the virus within the host insect [25]. Recent evidence suggests that FGF initiates a cascade of events that may accelerate the establishment of systemic infections [26]. In our study, *fgf* was not found in the CapoNPV genome.

#### CapoNPV lacks *ac30*, a gene specific to Group I

In the previous report, 11 genes (*gp64*, *tyrosine phosphatase* gene (*ptp*), *ie2*, *odv-e26*, *ac5*, *ac30*, *ac73*, *ac72*, *ac114*, *ac124*, *ac132*) have been identified as specific to Group I viruses and are absent from all other baculoviruses [13]. These genes might have had an evolutionary role in the emergence of Group I viruses [13, 27]. Notably absent from CapoNPV is a homologue to *ac30*. This gene seems to be nonessential because deletion thereof did not affect the production of BmNPV [28]. Interestingly, CyunNPV, a member of Group I also lacks *ac30* (data not shown).

#### CapoNPV lacks *lef-7*, a gene involved in DNA replication

*lef-7* had stimulatory effects on transient DNA replication [29]. It is present in all previously identified Group I viruses, several Group II viruses and many betabaculoviruses. Deletion of *lef*-*7* from AcMNPV had no impact on virus infection in Tn368 cells, but in SF21 and SE1c cells the viral DNA replication was reduced to only 10% of the wild-type virus [30], suggesting the function of LEF7 is host dependent. *lef-7* was also found to be involved in the regulation of the DNA damage response (DDR). Deletion of *lef-7* from the AcMNPV genome caused the activation of the DDR, and progeny infectious virus decreased about 99% [31]. CapoNPV is the first reported group I virus that does not contain a *lef*-*7* gene.

#### CapoNPV lacks ODV-E66, a structure protein of ODV involved in oral infection

ODV-E66 was identified as a component of ODV envelopes [32]. AcMNPV ODV-E66 was shown to have chondroitinase activity [33] and its crystal structure was determined [34]. It was suggested that ODV-E66 may function in midgut infection by degrading the peritrophic membrane, which contains a low level of chondroitin sulfate [33]. In fact deletion of *odv-e66* in AcMNPV increased the oral infection dose about 1000 times while did not changed the infectivity of BV, suggesting ODV-E66 is an important oral infectivity factor [35]. *Odv-e66* is present in most alphabaculoviruses and betabaculoviruses, however, it was not found in CapoNPV genome.

### F-like protein

A characteristic feature of Group I viruses is the presence of GP64 and the loss of fusion function of F. Except for gammabaculoviruses and Group I viruses, the F protein functions as the envelope fusion protein of BV. In AcMNPV, the F-like protein is also associated with BV membranes and its deletion from the genome results in infectious virus with titers similar to the parental virus in cell cultures, but the time to kill larvae is somewhat extended [36].

Previous studies showed the importance of the furin cleavage site in the fusion process. Furin protease digests F into two components, a small N-terminus membrane-anchored F2 and a large domain F1 at the C-terminus. Both are needed for viral-host membrane fusion [7, 37]. The F-like protein in Group I viruses lacks the furin cleavage site and, therefore, lost its fusion function. Instead, GP64 functions as an efficient envelope fusion protein [38–40].

**Fig 5:**
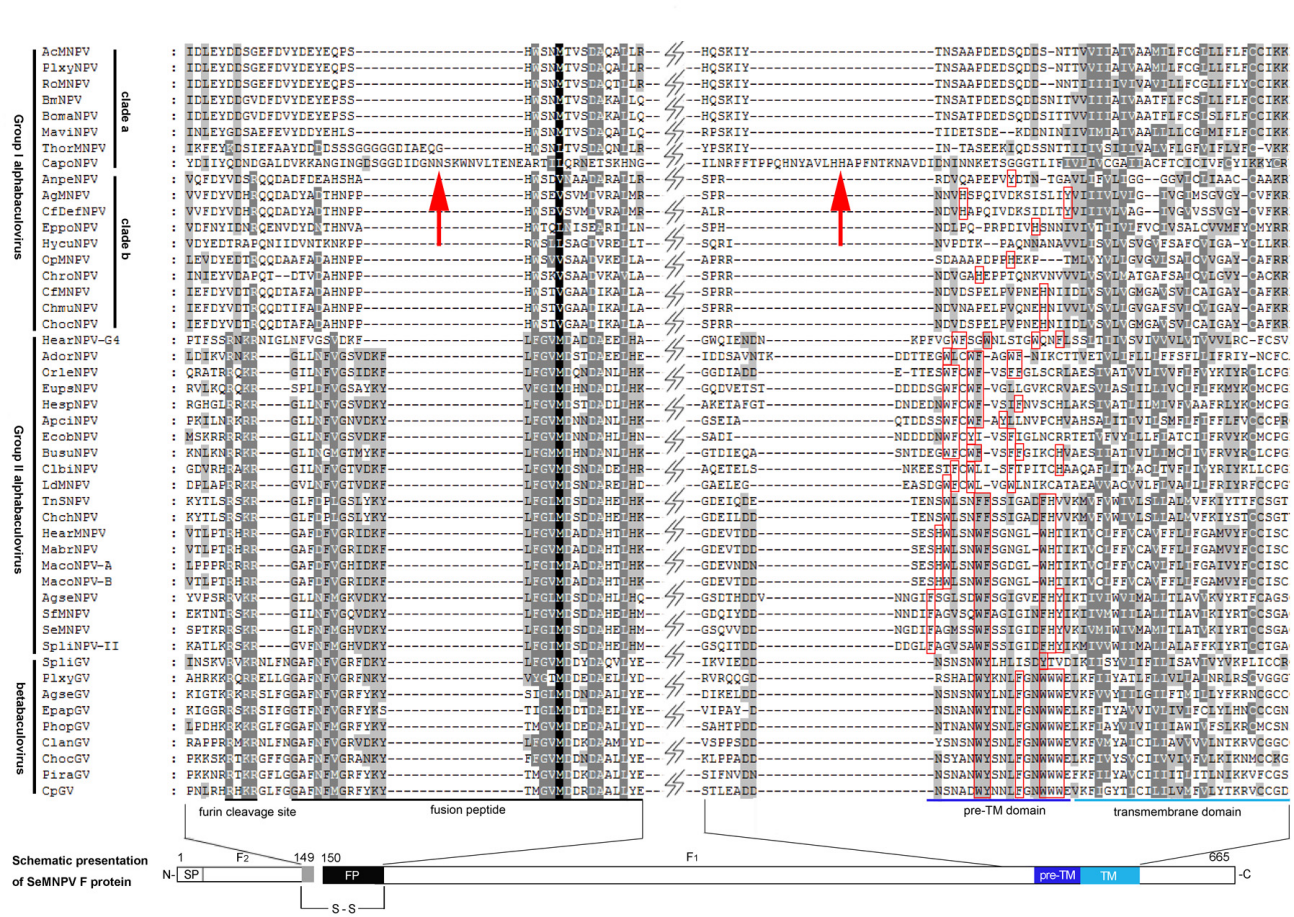
The amino acid alignment of F and F-like proteins. The alignment was performed using ClusterW method. A schematic figure of SeMNPV F protein was adapted from a previous publication [45] and is shown at the bottom, and two enlarged regions with sequence alignments are also shown. Viral names and categories are on the left. The predicted regions of furin cleavage site, fusion peptide, pre-TM and transmembrane domains are indicated below the alignment. The red square shows the aromatic amino acids (F, Y, W and H) in the pre-TM region. The arrows point to the insertion regions in CapoNPV. Black background shows greater than 80% identity among compared regions, dark gray and light gray shows greater than 50% and 30% identity, respectively.

In our study on F-like protein in CapoNPV, an insertion was found in the region equivalent to the fusion peptide (Fig 5). We also found another stretch of amino acids are inserted ahead of the pre-transmembrane domain (pre-TM) of CapoNPV (Fig 5). Sometimes, pre-TM domain, which is rich in aromatic amino acids, plays an important role in membrane fusion [41–44]. Similar insertions into the fusion peptide region and the pre-TM were also found in CyunNPV (data not shown).

According to phylogeny (Fig 2), CapoNPV evolved relatively earlier than other Group I alphabaculoviruses. Thysanoplusia orichalcea nucleopolyhedrovirus (ThorNPV), another relatively early member of Group I (Fig 2) also has an insertion at the fusion peptide region (Fig 5). The change of viral fusion ability mediated by the presence of GP64 and the inactivation of F are considered critical events in the origination of Group I [13]. Our results provide new evidence in the understanding of the process of F inactivation and, therefore, the early evolutionary events of Group I alphabaculoviruses.

## Materials and Methods

### Viral DNA Extraction

CapoNPV infected *Catopsilia pomona* larvae have been preserved in the ‘‘Chinese general virus collection center” (CGVCC) with collection number IVCAS 1.0228. OBs were purified from homogenized larvae by differential centrifugation [46] and DNA was extracted as described previously [47].

### Sequencing and Bioinformatics Analyses

The genome of CapoNPV was sequenced with the Roche 454 GS FLX+ system by using a shotgun strategy. The determined nucleotide sequences were assembled with GS De Novo Assembler software version 2.7. The complete genome sequence and annotation information were submitted to GenBank (accession number: KU565883).

Putative ORFs were analyzed using the FGENESV0 program (http://www.softberry.com/berry.phtml) [48] and the NCBI ORF Finder (http://www.ncbi.nlm.nih.gov/gorf/gorf.html). ORFs potentially encoding more than 50 amino acids were designated as putative genes with minimal overlaps. Gene parity plot analysis was performed as previously described [17, 49]. The Tandem Repeats Finder (http://tandem.bu.edu/trf/trf.html) was used to locate *hr*s. Gene annotation, comparisons were done with the aid of NCBI BLAST algorithm (http://blast.ncbi.nlm.nih.gov/Blast.cgi).

### Phylogenetic Analysis

A phylogenetic tree was generated based on amino acid sequences encoded by the 37 core genes from CapoNPV and that of the other 79 reference genome sequences of baculoviruses in NCBI (S1 Table). All the sequences were joined together in the same order and the alignments were generated using muscle method of MEGA6 with default settings. A phylogenetic tree was constructed by MEGA6 using Maximum Likelihood method based on the JTT matrix-based model [–50]. Phylogeny tested by Bootstrap method with a value of 1000 [51].

## Supporting Information

S1 Table Basic information of all sequenced baculovirus genomes in GenBank. (DOCX)

S2 Table The genome annotation of CapoNPV. (XLSX)

## References

[1] Lauzon HA, Lucarotti CJ, Krell PJ, Feng Q, Retnakaran A, Arif BM. Sequence and organization of the Neodiprion lecontei nucleopolyhedrovirus genome. J Virol. 2004;78(13):7023–35. 10.1128/JVI.78.13.7023-7035.2004 15194779 PMC421645

[2] Hayakawa T, Ko R, Okano K, Seong SI, Goto C, Maeda S. Sequence analysis of the Xestia c-nigrum granulovirus genome. Virology. 1999;262(2):277–97. 10.1006/viro.1999.9894 . 10502508

[3] Keddie BA, Aponte GW, Volkman LE. The pathway of infection of Autographa californica nuclear polyhedrosis virus in an insect host. Science. 1989;243(4899):1728–30. . 2648574 10.1126/science.2648574

[4] Jehle JA, Blissard GW, Bonning BC, Cory JS, Herniou EA, Rohrmann GF, et al On the classification and nomenclature of baculoviruses: a proposal for revision. Archives of virology. 2006;151(7):1257–66. 10.1007/s00705-006-0763-6 . 16648963

[5] Carstens EB, Ball LA. Ratification vote on taxonomic proposals to the International Committee on Taxonomy of Viruses (2008). Archives of virology. 2009;154(7):1181–8. 10.1007/s00705-009-0400-2 . 19495937 PMC7086627

[6] Hefferon KL, Oomens AG, Monsma SA, Finnerty CM, Blissard GW. Host cell receptor binding by baculovirus GP64 and kinetics of virion entry. Virology. 1999;258(2):455–68. 10.1006/viro.1999.9758 . 10366584

[7] WF IJ, Westenberg M, Goldbach RW, Blissard GW, Vlak JM, Zuidema D. A novel baculovirus envelope fusion protein with a proprotein convertase cleavage site. Virology. 2000;275(1):30–41. 10.1006/viro.2000.0483 . 11017785

[8] Monsma SA, Oomens AG, Blissard GW. The GP64 envelope fusion protein is an essential baculovirus protein required for cell-to-cell transmission of infection. Journal of virology. 1996;70(7):4607–16. 8676487 10.1128/jvi.70.7.4607-4616.1996 PMC190397

[9] Jehle JA, Lange M, Wang H, Hu Z, Wang Y, Hauschild R. Molecular identification and phylogenetic analysis of baculoviruses from Lepidoptera. Virology. 2006;346(1):180–93. 10.1016/j.virol.2005.10.032 . 16313938

[10] Garavaglia MJ, Miele SA, Iserte JA, Belaich MN, Ghiringhelli PD. The ac53, ac78, ac101, and ac103 genes are newly discovered core genes in the family Baculoviridae. Journal of virology. 2012;86(22):12069–79. Epub 2012/08/31. 10.1128/jvi.01873-12 ; PubMed Central PMCID: PMCPmc3486445. 22933288 PMC3486445

[11] Herniou EA, Olszewski JA, Cory JS, O'Reilly DR. The genome sequence and evolution of baculoviruses. Annual review of entomology. 2003;48:211–34. 10.1146/annurev.ento.48.091801.112756 . 12414741

[12] Chen YR, Wu CY, Lee ST, Wu YJ, Lo CF, Tsai MF, et al Genomic and host range studies of Maruca vitrata nucleopolyhedrovirus. J Gen Virol. 2008;89(Pt 9):2315–30. 10.1099/vir.0.2008/001412-0 . 18753242

[13] Jiang Y, Deng F, Rayner S, Wang H, Hu Z. Evidence of a major role of GP64 in group I alphabaculovirus evolution. Virus Res. 2009;142(1–2):85–91. 10.1016/j.virusres.2009.01.015 . 19428740

[14] Zhang L, Zhou S, Zhang Y. Catopsilia pomona seriously harm to plants of the genus Cassia. Plant Protection. 2003;1.

[15] Yang M, Lin Y, Liang S, Yuan A, Sun F. Preliminary Study on Catopsilia pomana NPV. Forest Research. 1993;6(2).

[16] Hu ZH, Arif BM, Jin F, Martens JW, Chen XW, Sun JS, et al Distinct gene arrangement in the Buzura suppressaria single-nucleocapsid nucleopolyhedrovirus genome. The Journal of general virology. 1998;79 (Pt 11):2841–51. . 9820162 10.1099/0022-1317-79-11-2841

[17] Kool M, Voeten JT, Goldbach RW, Tramper J, Vlak JM. Identification of seven putative origins of Autographa californica multiple nucleocapsid nuclear polyhedrosis virus DNA replication. The Journal of general virology. 1993;74 (Pt 12):2661–8. . 8277271 10.1099/0022-1317-74-12-2661

[18] Hilton S, Winstanley D. The origins of replication of granuloviruses. Archives of virology. 2008;153(8):1527–35. 10.1007/s00705-008-0148-0 . 18612587

[19] Carstens EB, Wu Y. No single homologous repeat region is essential for DNA replication of the baculovirus Autographa californica multiple nucleopolyhedrovirus. The Journal of general virology. 2007;88(Pt 1):114–22. 10.1099/vir.0.82384-0 . 17170443

[20] Guarino LA, Gonzalez MA, Summers MD. Complete Sequence and Enhancer Function of the Homologous DNA Regions of Autographa californica Nuclear Polyhedrosis Virus. Journal of virology. 1986;60(1):224–9. 16789259 10.1128/jvi.60.1.224-229.1986 PMC253920

[21] Choi J, Guarino LA. The baculovirus transactivator IE1 binds to viral enhancer elements in the absence of insect cell factors. Journal of virology. 1995;69(7):4548–51. 7769721 10.1128/jvi.69.7.4548-4551.1995 PMC189203

[22] Rodems SM, Friesen PD. Transcriptional enhancer activity of hr5 requires dual-palindrome half sites that mediate binding of a dimeric form of the baculovirus transregulator IE1. Journal of virology. 1995;69(9):5368–75. 7636981 10.1128/jvi.69.9.5368-5375.1995 PMC189379

[23] Detvisitsakun C, Berretta MF, Lehiy C, Passarelli AL. Stimulation of cell motility by a viral fibroblast growth factor homolog: proposal for a role in viral pathogenesis. Virology. 2005;336(2):308–17. 10.1016/j.virol.2005.03.013 . 15892971

[24] Detvisitsakun C, Hutfless EL, Berretta MF, Passarelli AL. Analysis of a baculovirus lacking a functional viral fibroblast growth factor homolog. Virology. 2006;346(2):258–65. Epub 2006/02/16. 10.1016/j.virol.2006.01.016 . 16476460

[25] Detvisitsakun C, Cain EL, Passarelli AL. The Autographa californica M nucleopolyhedrovirus fibroblast growth factor accelerates host mortality. Virology. 2007;365(1):70–8. Epub 2007/04/27. 10.1016/j.virol.2007.03.027 . 17459443

[26] Means JC, Passarelli AL. Viral fibroblast growth factor, matrix metalloproteases, and caspases are associated with enhancing systemic infection by baculoviruses. Proceedings of the National Academy of Sciences of the United States of America. 2010;107(21):9825–30. Epub 2010/05/12. 10.1073/pnas.0913582107 ; PubMed Central PMCID: PMCPmc2906863. 20457917 PMC2906863

[27] Herniou EA, Luque T, Chen X, Vlak JM, Winstanley D, Cory JS, et al Use of whole genome sequence data to infer baculovirus phylogeny. Journal of virology. 2001;75(17):8117–26. 11483757 10.1128/JVI.75.17.8117-8126.2001 PMC115056

[28] Huang J, Hao B, Deng F, Sun X, Wang H, Hu Z. Open reading frame Bm21 of Bombyx mori nucleopolyhedrovirus is not essential for virus replication in vitro, but its deletion extends the median survival time of infected larvae. The Journal of general virology. 2008;89(Pt 4):922–30. 10.1099/vir.0.83504-0 . 18343833

[29] Lu A, Miller LK. The roles of eighteen baculovirus late expression factor genes in transcription and DNA replication. Journal of virology. 1995;69(2):975–82. PMC188666. 7815565 10.1128/jvi.69.2.975-982.1995 PMC188666

[30] Chen C-J, Thiem SM. Differential Infectivity of Two Autographa californica Nucleopolyhedrovirus Mutants on Three Permissive Cell Lines Is the Result of lef-7 Deletion. Virology. 1997;227(1):88–95. 10.1006/viro.1996.8341 9007061

[31] Mitchell JK, Byers NM, Friesen PD. Baculovirus F-Box Protein LEF-7 Modifies the Host DNA Damage Response To Enhance Virus Multiplication. Journal of virology. 2013;87(23):12592–9. 10.1128/JVI.02501-13 PMC3838121. 24027328 PMC3838121

[32] Hong T, Braunagel SC, Summers MD. Transcription, Translation, and Cellular Localization of PDV-E66: A Structural Protein of the PDV Envelope of Autographa californica Nuclear Polyhedrosis Virus. Virology. 1994;204(1):210–22. 10.1006/viro.1994.1525 8091653

[33] Sugiura N, Setoyama Y, Chiba M, Kimata K, Watanabe H. Baculovirus Envelope Protein ODV-E66 Is a Novel Chondroitinase with Distinct Substrate Specificity. The Journal of biological chemistry. 2011;286(33):29026–34. 10.1074/jbc.M111.251157 PMC3190710. 21715327 PMC3190710

[34] Kawaguchi Y, Sugiura N, Onishi M, Kimata K, Kimura M, Kakuta Y. Crystallization and X-ray diffraction analysis of chondroitin lyase from baculovirus: envelope protein ODV-E66. Acta Crystallographica Section F: Structural Biology and Crystallization Communications. 2012;68(Pt 2):190–2. 10.1107/S1744309111053164 PMC3274400. 22297996 PMC3274400

[35] Xiang X, Chen L, Hu X, Yu S, Yang R, Wu X. Autographa californica multiple nucleopolyhedrovirus odv-e66 is an essential gene required for oral infectivity. Virus Research. 2011;158(1–2):72–8. 10.1016/j.virusres.2011.03.012 21440017

[36] Lung OY, Cruz-Alvarez M, Blissard GW. Ac23, an envelope fusion protein homolog in the baculovirus Autographa californica multicapsid nucleopolyhedrovirus, is a viral pathogenicity factor. Journal of virology. 2003;77(1):328–39. Epub 2002/12/13. ; PubMed Central PMCID: PMCPmc140606. 12477838 10.1128/JVI.77.1.328-339.2003 PMC140606

[37] Westenberg M, Wang H, WF IJ, Goldbach RW, Vlak JM, Zuidema D. Furin is involved in baculovirus envelope fusion protein activation. Journal of virology. 2002;76(1):178–84. Epub 2001/12/12. ; PubMed Central PMCID: PMCPmc135720. 11739683 10.1128/JVI.76.1.178-184.2002 PMC135720

[38] Hohmann AW, Faulkner P. Monoclonal antibodies to baculovirus structural proteins: determination of specificities by Western blot analysis. Virology. 1983;125(2):432–44. . 6340331 10.1016/0042-6822(83)90214-3

[39] Whitford M, Stewart S, Kuzio J, Faulkner P. Identification and sequence analysis of a gene encoding gp67, an abundant envelope glycoprotein of the baculovirus Autographa californica nuclear polyhedrosis virus. Journal of virology. 1989;63(3):1393–9. 2644449 10.1128/jvi.63.3.1393-1399.1989 PMC247838

[40] Volkman LE. The 64K envelope protein of budded Autographa californica nuclear polyhedrosis virus. Current topics in microbiology and immunology. 1986;131:103–18. . 3545692 10.1007/978-3-642-71589-1_6

[41] Li Z, Blissard GW. The pre-transmembrane domain of the Autographa californica multicapsid nucleopolyhedrovirus GP64 protein is critical for membrane fusion and virus infectivity. Journal of virology. 2009;83(21):10993–1004. Epub 2009/08/21. 10.1128/jvi.01085-09 ; PubMed Central PMCID: PMCPmc2772811. 19692475 PMC2772811

[42] Saez-Cirion A, Gomara MJ, Agirre A, Nieva JL. Pre-transmembrane sequence of Ebola glycoprotein. Interfacial hydrophobicity distribution and interaction with membranes. FEBS letters. 2003;533(1–3):47–53. Epub 2002/12/31. . 12505157 10.1016/s0014-5793(02)03747-x

[43] Guillen J, Moreno MR, Perez-Berna AJ, Bernabeu A, Villalain J. Interaction of a peptide from the pre-transmembrane domain of the severe acute respiratory syndrome coronavirus spike protein with phospholipid membranes. The journal of physical chemistry B. 2007;111(49):13714–25. Epub 2007/11/21. 10.1021/jp073675y . 18020324

[44] Lorizate M, Huarte N, Saez-Cirion A, Nieva JL. Interfacial pre-transmembrane domains in viral proteins promoting membrane fusion and fission. Biochimica et biophysica acta. 2008;1778(7–8):1624–39. Epub 2008/01/29. 10.1016/j.bbamem.2007.12.018 . 18222166 PMC7094410

[45] Westenberg M, Veenman F, Roode EC, Goldbach RW, Vlak JM, Zuidema D. Functional analysis of the putative fusion domain of the baculovirus envelope fusion protein F. Journal of virology. 2004;78(13):6946–54. 10.1128/JVI.78.13.6946-6954.2004 15194771 PMC421653

[46] Huang JP TW, Shu HQ. The research of Cyclophragma undans nucleopolyhedrovirus. journal of central south forestry institute. 1983;3(2):136–42.

[47] Hu ZH, Arif BM, Sun JS, Chen XW, Zuidema D, Goldbach RW, et al Genetic organization of the HindIII-I region of the single-nucleocapsid nucleopolyhedrovirus of Buzura suppressaria. Virus research. 1998;55(1):71–82. . 9712513 10.1016/s0168-1702(98)00029-x

[48] Solovyev VV, Salamov AA. INFOGENE: a database of known gene structures and predicted genes and proteins in sequences of genome sequencing projects. Nucleic acids research. 1999;27(1):248–50. Epub 1998/12/10. ; PubMed Central PMCID: PMCPmc148147. 9847192 10.1093/nar/27.1.248 PMC148147

[49] Zhu Z, Yin F, Liu X, Hou D, Wang J, Zhang L, et al Genome sequence and analysis of Buzura suppressaria nucleopolyhedrovirus: a group II Alphabaculovirus. PLoS One. 2014;9(1):e86450 10.1371/journal.pone.0086450 24475121 PMC3901692

[50] Tamura K, Stecher G, Peterson D, Filipski A, Kumar S. MEGA6: Molecular Evolutionary Genetics Analysis version 6.0. Mol Biol Evol. 2013;30(12):2725–9. 10.1093/molbev/mst197 24132122 PMC3840312

[51] Sanderson MJ, Wojciechowski MF. Improved bootstrap confidence limits in large-scale phylogenies, with an example from Neo-Astragalus (Leguminosae). Systematic biology. 2000;49(4):671–85. Epub 2002/07/16. . 12116433 10.1080/106351500750049761

