# Supplementary material for: Genome Sequencing and Analysis of Catopsilia pomona nucleopolyhedrovirus: A Distinct Species in Group I Alphabaculovirus
Source: PLoS One. 2016 May 11;11(5):e0155134. doi: 10.1371/journal.pone.0155134 (PMC4864199; doi:10.1371/journal.pone.0155134)
Supplement: S1 Table — (DOCX) [file pone.0155134.s001.docx]

**S1 Table**. Basic information of all sequenced baculovirus genomes in GenBank*.

| Genus | Virus | Abbreviation | Accession  no. | Length (bp) | Orfs | *hr*s | *bro*s | G+C (%) | Reference |
| --- | --- | --- | --- | --- | --- | --- | --- | --- | --- |
| *Alphabaculovirus* (Group I) | Antheraea pernyi NPV | AnpeNPV | NC_008035 | 126629 | 147 | 3 | 2 | 53.5 | [1] |
|  | Anticarsia gemmatalis NPV | AngeNPV | NC_008520 | 132239 | 158 | 9 | 7 | 44.5 | [2] |
|  | Autographa californica MNPV | AcMNPV | NC_001623 | 133894 | 156 | 9 | 1 | 40.7 | [3] |
|  | Bombyx mandarina NPV | BomaNPV | NC_012672 | 126770 | 141 | 7 | 3 | 40.2 | [4] |
|  | Bombyx mori NPV | BmNPV | NC_001962 | 128413 | 143 | 7 | 5 | 40.4 | [5] |
|  | Catopsilia Pomona NPV | CapoNPV | KU565883 | 128058 | 131 | 8 | 1 | 39.7 | this paper |
|  | Choristoneura fumiferana DEF MNPV | CfDEFMNPV | NC_005137 | 131160 | 149 | 13 | 4 | 45.8 | [6] |
|  | Choristoneura fumiferana MNPV | CfMNPV | NC_004778 | 129593 | 146 | 5 | 1 | 50.1 | [7] |
|  | Choristoneura murinana NPV | ChmuNPV | NC_023177 | 124688 | 147 | 2 | 1 | 50 | [8] |
|  | Choristoneura occidentalis NPV | ChocNPV | NC_021925 | 128446 | 148 | 5 | 2 | 50.1 | [9] |
|  | Choristoneura rosaceana NPV | ChroNPV | NC_021924 | 129052 | 149 | 3 | 2 | 48.6 | [9] |
|  | Condylorrhiza vestigialis MNPV | CoveMNPV | NC_026430 | 125767 | 138 | 4 | 9 | 42.9 | [10] |
|  | Ecotropis obliqua NPV | EcobNPV | NC_008586 | 131204 | 126 | 3 | 2 | 37.6 | [11] |
|  | Hyphantria cunea NPV | HycuNPV | NC_007767 | 132959 | 148 | 6 | 5 | 45.5 | [12] |
|  | Maruca vitrata MNPV | MaviMNPV | NC_008725 | 111953 | 126 | 5 | 0 | 38.6 | [13] |
|  | Orgyia pseudotsugata MNPV | OpMNPV | NC_001875 | 131995 | 152 | 5 | 1 | 55.1 | [14] |
|  | Philosamia cynthia ricini NPV | PhcyNPV | JX404026 | 125376 | 138 | 6 | 2 | 53.7 | [15] |
|  | Plutella xylostellaMNPV | PlxyMNPV | NC_008349 | 134417 | 152 | 9 | 2 | 40.7 | [16] |
|  | Rachiplusia ou MNPV | RoMNPV | NC_004323 | 131526 | 149 | 9 | 0 | 39.1 | [17] |
|  | Thysanoplusia orichalcea NPV | ThorNPV | NC_019945 | 132978 | 145 | 6 | 2 | 39.5 | [18] |
| *Alphabaculovirus* (Group II) | Adoxophyes honmai NPV | AdhoNPV | NC_004690 | 113220 | 125 | 4 | 4 | 35.6 | [19] |
|  | Adoxophyes orana NPV | AdorNPV | NC_011423 | 111724 | 121 | 4 | 3 | 35 | [20] |
|  | Agrotis ipsilon MNPV | AgipMNPV | NC_011345 | 155122 | 163 | 7 | 5 | 48.6 | [21] |
|  | Agrotis segetum NPV | AgseNPV | NC_007921 | 147544 | 153 | 5 | 4 | 45.7 | [22] |
|  | Agrotis segetum NPV B | AgseNPV-B | NC_025960 | 148981 | 150 | 6 | 2 | 45.7 | [23] |
|  | Apocheima cinerarium NPV | ApciNPV | NC_018504 | 123876 | 117 | 4 | 1 | 33.4 | [24] |
|  | Buzura suppressaria NPV | BusuNPV | NC_023442 | 120420 | 127 | 0 | 3 | 36.8 | [25] |
|  | Chrysodeixis chalcites NPV | ChchNPV | NC_007151 | 149622 | 151 | 0 | 4 | 39 | [26] |
|  | Clanis bilineata NPV | ClbiNPV | NC_008293 | 135454 | 129 | 0 | 3 | 37.7 | [27] |
|  | Epiphyas postvittana NPV | EppoNPV | NC_003083 | 118584 | 136 | 5 | 1 | 40.7 | [28] |
|  | Euproctis pseudoconspersa NPV | EupsNPV | NC_012639 | 141291 | 139 | 4 | 2 | 40.3 | [29] |
|  | Helicoverpa armigeraSNPV AC53 | HaSNPV-AC53 | NC_024688 | 130442 | 138 | 0 | 3 | 39.1 | [30] |
|  | Helicoverpa armigera MNPV | HearMNPV | NC_011615 | 154196 | 162 | 4 | 6 | 40.1 | [31] |
|  | Helicoverpa armigera NPV | HearNPV | NC_003094 | 130759 | 137 | 5 | 3 | 38.9 | [32] |
|  | Helicoverpa armigera NPV G4 | HearNPV-G4 | NC_002654 | 131405 | 135 | 5 | 3 | 39 | [33] |
|  | Helicoverpa armigera NPV NNg1 | HearNPV-NNg1 | NC_011354 | 132425 | 143 | 5 | 4 | 39.2 | [34] |
|  | Helicoverpa zea SNPV | HzSNPV | NC_003349 | 130869 | 139 | 5 | 3 | 39.1 | [35] |
|  | Hemileuca sp. NPV | HespNPV | NC_021923 | 140633 | 137 | 3 | 2 | 38.1 | [36] |
|  | Lambdina fiscellaria NPV | LafiNPV | NC_026922 | 157977 | 137 | 2 | 0 | 43.7 | [37] |
|  | Leucania separata NPV | LeseNPV | NC_008348 | 168041 | 169 | 8 | 10 | 48.6 | [38] |
|  | Lymantria dispar MNPV | LdMNPV | NC_001973 | 161046 | 164 | 13 | 16 | 57.5 | [39] |
|  | Lymantria xylina MNPV | LdxyMNPV | NC_013953 | 156344 | 157 | 13 | 14 | 53.5 | [40] |
|  | Mamestra brassicae MNPV | MabrMNPV | NC_023681 | 152710 | 159 | 4 | 6 | 39.9 | [41] |
|  | Mamestra configurata NPV-A | MacoNPV-A | NC_003529 | 155060 | 169 | 4 | 8 | 41.7 | [42] |
|  | Mamestra configurata NPV-B | MacoNPV-B | NC_004117 | 158482 | 168 | 4 | 7 | 40 | [43] |
|  | Orgyia leucostigma NPV | OrleNPV | NC_010276 | 156179 | 135 | 3 | 5 | 39.9 | [44] |
|  | Peridroma NPV | PespNPV | NC_024625 | 151109 | 139 | 2 | 6 | 53.2 | [45] |
|  | Perigonia lusca single NPV | PeluNPV | NC_027923 | 132831 | 145 | 2 | 1 | 40 | NP |
|  | Pseudoplusia includens SNPV | PsinNPV | NC_026268 | 139132 | 141 | 0 | 2 | 39.3 | [46] |
|  | Spodoptera exigua MNPV | SeMNPV | NC_002169 | 135611 | 139 | 6 | 0 | 43.8 | [47] |
|  | Spodoptera frugiperda MNPV virus | SfMNPV | NC_009011 | 131331 | 143 | 8 | 1 | 40.2 | [48] |
|  | Spodoptera litura NPV | SpltNPV | NC_003102 | 139342 | 141 | 17 | 2 | 42.8 | [49] |
|  | Spodoptera litura NPV II | SpltNPV-II | NC_011616 | 148634 | 147 | 7 | 2 | 45 | NP |
|  | Sucra jujuba NPV | SujuNPV | KJ676450 | 135952 | 131 | 7 | 4 | 38.7 | [50] |
|  | Trichoplusia ni SNPV | TnSNPV | NC_007383 | 134394 | 145 | 0 | 2 | 39 | [51] |
| *Betabaculovirus* | Adoxophyes orana granulovirus | AdorGV | NC_005038 | 99657 | 119 | 0 | 0 | 34.5 | [52] |
|  | Agrotis segetum granulovirus | AgseGV | NC_005839 | 131680 | 132 | 0 | 0 | 37.3 | NP |
|  | Clostera anastomosis GV isolate Henan | ClasGV-A | NC_022646 | 101818 | 122 | 0 | 0 | 46.7 | [53] |
|  | Clostera anastomosis granulovirus-B | ClasGV-B | KR091910 | 107439 | 123 | 0 | 0 | 37.8 | [54] |
|  | Cnaphalocrocis medinalis GV | CnmeGV | - | 111246 | 118 | 0 | 3 | 35 | [55] |
|  | Choristoneura occidentalis GV | ChocGV | NC_008168 | 104710 | 116 | 5 | 0 | 32.7 | [56] |
|  | Clostera anachoreta granulovirus | ClanGV | NC_015398 | 101487 | 123 | 4 | 0 | 44.4 | [57] |
|  | Cydia pomonella granulovirus | CpGV | NC_002816 | 123500 | 143 | 0 | 1 | 45.3 | [58] |
|  | Cryptophlebia leucotreta granulovirus | CrleGV | NC_005068 | 110907 | 128 | 3 | 0 | 32.4 | [59] |
|  | Diatraea saccharalis granulovirus | DisaGV | NC_028491 | 98392 | 125 | 7 | 0 | 35 | [60] |
|  | Epinotia aporema granulovirus | EpapGV | NC_018875 | 119082 | 132 | 16 | 0 | 41.5 | [61] |
|  | Erinnyis ello granulovirus | ErelGV | NC_025257 | 102759 | 130 | 5 | 0 | 38.7 | [62] |
|  | Helicoverpa armigera granulovirus | HearGV | NC_010240 | 169794 | 179 | 9 | 10 | 40.8 | [63] |
|  | Phthorimaea operculella granulovirus | PhopGV | NC_004062 | 119217 | 130 | 12 | 1 | 35.7 | [64] |
|  | Plutella xylostella granulovirus | PlxyGV | NC_002593 | 100999 | 120 | 4 | 0 | 40.7 | [65] |
|  | Pieris rapae granulovirus | PrGV | NC_013797 | 108592 | 120 | 0 | 0 | 33.2 | [66] |
|  | Pseudaletia unipuncta granulovirus | PsunGV | NC_013772 | 176677 | 183 | 9 | 11 | 39.8 | NP |
|  | Spodoptera frugiperda GV isolateVG008 | SpfrGV | NC_026511 | 140913 | 146 | 8 | 7 | 46.2 | [67] |
|  | Spodoptera litura granulovirus | SpliGV | NC_009503 | 124121 | 136 | 0 | 6 | 38.8 | [68] |
|  | Xestia c-nigrum granulovirus | XcGV | NC_002331 | 178733 | 181 | 9 | 7 | 40.7 | [69] |
| *Gammabaculovirus* | Neodiprion abietis NPV | NeabNPV | NC_008252 | 84264 | 93 | 5 | 0 | 33.4 | [70] |
|  | Neodiprion lecontei NPV | NeleNPV | NC_005906 | 81755 | 89 | 0 | 0 | 33.3 | [71] |
|  | Neodiprion sertifer NPV | NeseNPV | NC_005905 | 86462 | 90 | 6 | 0 | 33.8 | [72] |
| *Deltabaculovirus* | Culex nigripalpus NPV | CuniNPV | NC_003084 | 108252 | 109 | 4 | 5 | 50.9 | [73] |

*Updated by Jan. 21^st^, 2016. NP means not published.

**Reference**

1. Nie ZM, Zhang ZF, Wang D, He PA, Jiang CY, et al. (2007) Complete sequence and organization of Antheraea pernyi nucleopolyhedrovirus, a dr-rich baculovirus. BMC Genomics 8: 248.

2. Oliveira JV, Wolff JL, Garcia-Maruniak A, Ribeiro BM, de Castro ME, et al. (2006) Genome of the most widely used viral biopesticide: Anticarsia gemmatalis multiple nucleopolyhedrovirus. J Gen Virol 87: 3233-3250.

3. Ayres MD, Howard SC, Kuzio J, Lopez-Ferber M, Possee RD (1994) The complete DNA sequence of Autographa californica nuclear polyhedrosis virus. Virology 202: 586-605.

4. Xu YP, Ye ZP, Niu CY, Bao YY, Wang WB, et al. (2010) Comparative analysis of the genomes of Bombyx mandarina and Bombyx mori nucleopolyhedroviruses. J Microbiol 48: 102-110.

5. Gomi S, Majima K, Maeda S (1999) Sequence analysis of the genome of Bombyx mori nucleopolyhedrovirus. J Gen Virol 80 ( Pt 5): 1323-1337.

6. Lauzon HA, Jamieson PB, Krell PJ, Arif BM (2005) Gene organization and sequencing of the Choristoneura fumiferana defective nucleopolyhedrovirus genome. J Gen Virol 86: 945-961.

7. de Jong JG, Lauzon HA, Dominy C, Poloumienko A, Carstens EB, et al. (2005) Analysis of the Choristoneura fumiferana nucleopolyhedrovirus genome. J Gen Virol 86: 929-943.

8. Rohrmann GF, Erlandson MA, Theilmann DA. (2014) Genome sequence of an alphabaculovirus isolated from Choristoneura murinana. Genome Announc. 2(1):e0113513. doi:10.1128/genomeA.01135-13.

9. Thumbi DK, Beliveau C, Cusson M, Lapointe R, Lucarotti CJ (2013) Comparative Genome Sequence Analysis of Choristoneura occidentalis Freeman and C. rosaceana Harris (Lepidoptera: Tortricidae) Alphabaculoviruses. PLoS One 8: e68968.

10. Maria Elita B. Castro, Zilda Maria A. Ribeiro, Ana Cláudia B. Santos, Marlinda L. Souza, Edilene B. Machado, Nilton J. Sousa, Flávio Moscardi, Identification of a new nucleopolyhedrovirus from naturally-infected Condylorrhiza vestigialis (Guenée) (Lepidoptera: Crambidae) larvae on poplar plantations in South Brazil, Journal of Invertebrate Pathology, Volume 102, Issue 2, October 2009, Pages 149-154, ISSN 0022-2011

11. Xiu-Cui Ma, Jin-Yan Shang, Zhang-Nv Yang, Yan-Yuan Bao, Qiang Xiao, Chuan-Xi Zhang, Genome sequence and organization of a nucleopolyhedrovirus that infects the tea looper caterpillar, Ectropis obliqua, Virology, Volume 360, Issue 1, 30 March 2007, Pages 235-246, ISSN 0042-6822, http://dx.doi.org/10.1016/j.virol.2006.10.024.

12. Ikeda M, Shikata M, Shirata N, Chaeychomsri S, Kobayashi M (2006) Gene organization and complete sequence of the Hyphantria cunea nucleopolyhedrovirus genome. J Gen Virol 87: 2549-2562.

13. Chen YR, Wu CY, Lee ST, Wu YJ, Lo CF, et al. (2008) Genomic and host range studies of Maruca vitrata nucleopolyhedrovirus. J Gen Virol 89: 2315-2330.

14. Ahrens CH, Russell RL, Funk CJ, Evans JT, Harwood SH, et al. (1997) The sequence of the Orgyia pseudotsugata multinucleocapsid nuclear polyhedrosis virus genome. Virology 229: 381-399.

15. Qian H, Zhang Y, Wu Y, Sun P, Zhu S, et al. (2013) Analysis of the genomic sequence of Philosamia cynthia nucleopolyhedrin virus and comparison with Antheraea pernyi nucleopolyhedrin virus. BMC Genomics 14: 115.

16. Harrison RL, Lynn DE (2007) Genomic sequence analysis of a nucleopolyhedrovirus isolated from the diamondback moth, Plutella xylostella. Virus Genes 35: 857-873.

17. Harrison RL, Bonning BC (2003) Comparative analysis of the genomes of Rachiplusia ou and Autographa californica multiple nucleopolyhedroviruses. J Gen Virol 84: 1827-1842.

18. Wang YS, Huang GH, Cheng XH, Wang X, Garretson TA, et al. (2012) Genome of Thysanoplusia orichalcea multiple nucleopolyhedrovirus lacks the superoxide dismutase gene. J Virol 86: 11948-11949.

19. Nakai M, Goto C, Kang W, Shikata M, Luque T, et al. (2003) Genome sequence and organization of a nucleopolyhedrovirus isolated from the smaller tea tortrix, Adoxophyes honmai. Virology 316: 171-183.

20. Hilton S, Winstanley D (2008) Genomic sequence and biological characterization of a nucleopolyhedrovirus isolated from the summer fruit tortrix, Adoxophyes orana. J Gen Virol 89: 2898-2908.

21. Harrison RL (2009) Genomic sequence analysis of the Illinois strain of the Agrotis ipsilon multiple nucleopolyhedrovirus. Virus Genes 38: 155-170.

22. Jakubowska AK, Peters SA, Ziemnicka J, Vlak JM, van Oers MM (2006) Genome sequence of an enhancin gene-rich nucleopolyhedrovirus (NPV) from Agrotis segetum: collinearity with Spodoptera exigua multiple NPV. J Gen Virol 87: 537-551.

23. Wennmann JT, Gueli Alletti G, Jehle JA. The genome sequence of Agrotis segetum nucleopolyhedrovirus B (AgseNPV-B) reveals a new baculovirus species within the Agrotis baculovirus complex. Virus Genes. 2014;50(2):260-76. doi: 10.1007/s11262-014-1148-7.

24. Thumbi DK, Eveleigh RJ, Lucarotti CJ, Lapointe R, Graham RI, Pavlik L, et al. Complete sequence, analysis and organization of the Orgyia leucostigma nucleopolyhedrovirus genome. Viruses. 2011;3(11):2301-27. doi: 10.3390/v3112301. PubMed PMID: 22163346; PubMed Central PMCID: PMCPMC3230853.

25. Zhu Z, Yin F, Liu X, Hou D, Wang J, Zhang L, et al. (2014) Genome Sequence and Analysis of Buzura suppressaria Nucleopolyhedrovirus: A Group II Alphabaculovirus. PLoS ONE 9(1): e86450. doi:10.1371/journal.pone.0086450

26. van Oers MM, Abma-Henkens MH, Herniou EA, de Groot JC, Peters S, et al. (2005) Genome sequence of Chrysodeixis chalcites nucleopolyhedrovirus, a baculovirus with two DNA photolyase genes. J Gen Virol 86: 2069-2080.

27. Zhu SY, Yi JP, Shen WD, Wang LQ, He HG, et al. (2009) Genomic sequence, organization and characteristics of a new nucleopolyhedrovirus isolated from Clanis bilineata larva. BMC Genomics 10: 91.

28. Hyink O, Dellow RA, Olsen MJ, Caradoc-Davies KMB, Drake K, Herniou EA, et al. Whole genome analysis of the Epiphyas postvittana nucleopolyhedrovirus. Journal of General Virology. 2002;83(4):957-71. doi: doi:10.1099/0022-1317-83-4-957.

29. Tang XD, Xiao Q, Ma XC, Zhu ZR, Zhang CX (2009) Morphology and genome of Euproctis pseudoconspersa nucleopolyhedrovirus. Virus Genes 38: 495-506.

30. Noune C, Hauxwell C. Complete Genome Sequences of Helicoverpa armigera Single Nucleopolyhedrovirus Strains AC53 and H25EA1 from Australia. Genome Announcements. 2015;3(5):e01083-15. doi: 10.1128/genomeA.01083-15. PubMed PMID: PMC4582581.

31 Tang P, Zhang H, Li Y, Han B, Wang G, Qin Q, et al. Genomic sequencing and analyses of HearMNPV--a new Multinucleocapsid nucleopolyhedrovirus isolated from Helicoverpa armigera. Virology journal. 2012;9:168. doi: 10.1186/1743-422X-9-168. PubMed PMID: 22913743; PubMed Central PMCID: PMC3545888.

32. Chen X, WF IJ, Tarchini R, Sun X, Sandbrink H, et al. (2001) The sequence of the Helicoverpa armigera single nucleocapsid nucleopolyhedrovirus genome. J Gen Virol 82: 241-257.

33. Zhang CX, Ma XC, Guo ZJ (2005) Comparison of the complete genome sequence between C1 and G4 isolates of the Helicoverpa armigera single nucleocapsid nucleopolyhedrovirus. Virology 333: 190-199.

34. Ogembo JG, Caoili BL, Shikata M, Chaeychomsri S, Kobayashi M, et al. (2009) Comparative genomic sequence analysis of novel Helicoverpa armigera nucleopolyhedrovirus (NPV) isolated from Kenya and three other previously sequenced Helicoverpa spp. NPVs. Virus Genes 39: 261-272.

35. Chen X, Zhang WJ, Wong J, Chun G, Lu A, et al. (2002) Comparative analysis of the complete genome sequences of Helicoverpa zea and Helicoverpa armigera single-nucleocapsid nucleopolyhedroviruses. J Gen Virol 83: 673-684.

36. Rohrmann GF, Erlandson MA, Theilmann DA (2013) The genome of a baculovirus isolated from Hemileuca sp. encodes a serpin ortholog. Virus Genes.

37. Rohrmann GF, Erlandson MA, Theilmann DA. Genome Sequence of an Alphabaculovirus Isolated from the Oak Looper, Lambdina fiscellaria, Contains a Putative 2-Kilobase-Pair Transposable Element Encoding a Transposase and a FLYWCH Domain-Containing Protein. Genome Announcements. 2015;3(3):e00186-15. doi: 10.1128/genomeA.00186-15. PubMed PMID: PMC4447894.

38. Xiao H, Qi Y (2007) Genome sequence of Leucania seperata nucleopolyhedrovirus. Virus Genes 35: 845-856.

39. Kuzio J, Pearson MN, Harwood SH, Funk CJ, Evans JT, et al. (1999) Sequence and analysis of the genome of a baculovirus pathogenic for Lymantria dispar. Virology 253: 17-34.

40. Nai YS, Wu CY, Wang TC, Chen YR, Lau WH, et al. (2010) Genomic sequencing and analyses of Lymantria xylina multiple nucleopolyhedrovirus. BMC Genomics 11: 116.

41. Choi JB, Heo WI, Shin TY, Bae SM, Kim WJ, et al. (2013) Complete genomic sequences and comparative analysis of Mamestra brassicae nucleopolyhedrovirus isolated in Korea. Virus Genes 47: 133-151.

42. Li Q, Donly C, Li L, Willis LG, Theilmann DA, et al. (2002) Sequence and organization of the Mamestra configurata nucleopolyhedrovirus genome. Virology 294: 106-121.

43. Li L, Li Q, Willis LG, Erlandson M, Theilmann DA, et al. (2005) Complete comparative genomic analysis of two field isolates of Mamestra configurata nucleopolyhedrovirus-A. J Gen Virol 86: 91-105.

44. Thumbi DK, Eveleigh RJ, Lucarotti CJ, Lapointe R, Graham RI, et al. (2011) Complete sequence, analysis and organization of the Orgyia leucostigma nucleopolyhedrovirus genome. Viruses 3: 2301-2327.

45. Rohrmann GF, Erlandson MA, Theilmann DA. A Distinct Group II Alphabaculovirus Isolated from a Peridroma Species. Genome Announcements. 2015;3(2):e00185-15. doi: 10.1128/genomeA.00185-15. PubMed PMID: PMC4384481.

46. Craveiro SR, Inglis PW, Togawa RC, Grynberg P, Melo FL, Ribeiro ZMA, et al. The genome sequence of Pseudoplusia includens single nucleopolyhedrovirus and an analysis of p26 gene evolution in the baculoviruses. BMC Genomics. 2015;16(1):1-13. doi: 10.1186/s12864-015-1323-9.

47. WF IJ, van Strien EA, Heldens JG, Broer R, Zuidema D, et al. (1999) Sequence and organization of the Spodoptera exigua multicapsid nucleopolyhedrovirus genome. J Gen Virol 80 ( Pt 12): 3289-3304.

48. Harrison RL, Puttler B, Popham HJ (2008) Genomic sequence analysis of a fast-killing isolate of Spodoptera frugiperda multiple nucleopolyhedrovirus. J Gen Virol 89: 775-790.

49. Pang Y, Yu J, Wang L, Hu X, Bao W, et al. (2001) Sequence analysis of the Spodoptera litura multicapsid nucleopolyhedrovirus genome. Virology 287: 391-404.

50. Liu X, Yin F, Zhu Z, Hou D, Wang J, Zhang L, et al. Genomic Sequencing and Analysis of Sucra jujuba Nucleopolyhedrovirus. PLoS ONE. 2014;9(10):e110023. doi: 10.1371/journal.pone.0110023. PubMed PMID: PMC4201490.

51. Leslie G. Willis, Robyn Siepp, Taryn M. Stewart, Martin A. Erlandson, David A. Theilmann, Sequence analysis of the complete genome of Trichoplusia ni single nucleopolyhedrovirus and the identification of a baculoviral photolyase gene, Virology, Volume 338, Issue 2, 1 August 2005, Pages 209-226, ISSN 0042-6822, http://dx.doi.org/10.1016/j.virol.2005.04.041.

52. Wormleaton S, Kuzio J, Winstanley D (2003) The complete sequence of the Adoxophyes orana granulovirus genome. Virology 311: 350-365.

53. Liang Z, Zhang X, Yin X, Cao S, Xu F (2011) Genomic sequencing and analysis of Clostera anachoreta granulovirus. Arch Virol 156: 1185-1198.

54. Yin F, Zhu Z, Liu X, Hou D, Wang J, Zhang L, et al. The Complete Genome of a New Betabaculovirus from Clostera anastomosis. PLoS ONE. 2015;10(7):e0132792. doi: 10.1371/journal.pone.0132792. PubMed PMID: PMC4500397.

55. Zhang S, Zhu Z, Sun S, Chen Q, Deng F, Yang K. Genome sequencing and analysis of a granulovirus isolated from the Asiatic rice leafroller, Cnaphalocrocis medinalis. Virologica Sinica. 2015;30(6):417-24. doi: 10.1007/s12250-015-3658-4.

56. Escasa SR, Lauzon HAM, Mathur AC, Krell PJ, Arif BM. Sequence analysis of the Choristoneura occidentalis granulovirus genome. Journal of General Virology. 2006;87(7):1917-33. doi: doi:10.1099/vir.0.81792-0.

57. Liang Z, Zhang X, Yin X, Song X, Shao X, et al. (2013) Comparative analysis of the genomes of Clostera anastomosis (L.) granulovirus and Clostera anachoreta granulovirus. Arch Virol 158: 2109-2114.

58. Luque T, Finch R, Crook N, O'Reilly DR, Winstanley D (2001) The complete sequence of the Cydia pomonella granulovirus genome. J Gen Virol 82: 2531-2547.

59. Lange M, Jehle JA (2003) The genome of the Cryptophlebia leucotreta granulovirus. Virology 317: 220-236.

60. Ardisson-Araujo DM, Melo FL, Clem RJ, Wolff JL, Ribeiro BM. A Betabaculovirus-Encoded gp64 Homolog Codes for a Functional Envelope Fusion Protein. J Virol. 2015;90(3):1668-72. doi: 10.1128/JVI.02491-15. PubMed PMID: 26537678.

61. Ferrelli ML, Salvador R, Biedma ME, Berretta MF, Haase S, et al. (2012) Genome of Epinotia aporema granulovirus (EpapGV), a polyorganotropic fast killing betabaculovirus with a novel thymidylate kinase gene. BMC Genomics 13: 548.

62. Ardisson-Araújo DMP, de Melo FL, Andrade MdS, Sihler W, Báo SN, Ribeiro BM, et al. Genome sequence of Erinnyis ello granulovirus (ErelGV), a natural cassava hornworm pesticide and the first sequenced sphingid-infecting betabaculovirus. BMC Genomics. 2014;15(1):856. doi: 10.1186/1471-2164-15-856. PubMed PMID: PMC4192325

63. Harrison RL, Popham HJ (2008) Genomic sequence analysis of a granulovirus isolated from the Old World bollworm, Helicoverpa armigera. Virus Genes 36: 565-581.

64. Taha A, Nour-El-Din A, Croizier L, Ferber ML, Croizier G (2000) Comparative analysis of the granulin regions of the Phthorimaea operculella and Spodoptera littoralis granuloviruses. Virus Genes 21: 147-155.

65. Hashimoto Y, Hayakawa T, Ueno Y, Fujita T, Sano Y, et al. (2000) Sequence analysis of the Plutella xylostella granulovirus genome. Virology 275: 358-372.

66. Zhang BQ, Cheng RL, Wang XF, Zhang CX (2012) The Genome of Pieris rapae Granulovirus. J Virol 86: 9544.

67. Cuartas PE, Barrera GP, Belaich MN, Barreto E, Ghiringhelli PD, Villamizar LF. The Complete Sequence of the First Spodoptera frugiperda Betabaculovirus Genome: A Natural Multiple Recombinant Virus. Viruses. 2015;7(1):394-421. doi: 10.3390/v7010394. PubMed PMID: PMC4306845.

68. Wang Y, Choi JY, Roh JY, Liu Q, Tao XY, et al. (2011) Genomic sequence analysis of granulovirus isolated from the tobacco cutworm, Spodoptera litura. PLoS One 6: e28163.

69. Hayakawa T, Ko R, Okano K, Seong SI, Goto C, et al. (1999) Sequence analysis of the Xestia c-nigrum granulovirus genome. Virology 262: 277-297.

70. Duffy SP, Young AM, Morin B, Lucarotti CJ, Koop BF, et al. (2006) Sequence analysis and organization of the Neodiprion abietis nucleopolyhedrovirus genome. J Virol 80: 6952-6963.

71. Lauzon HA, Lucarotti CJ, Krell PJ, Feng Q, Retnakaran A, et al. (2004) Sequence and organization of the Neodiprion lecontei nucleopolyhedrovirus genome. J Virol 78: 7023-7035.

72. Garcia-Maruniak A, Maruniak JE, Zanotto PM, Doumbouya AE, Liu JC, et al. (2004) Sequence analysis of the genome of the Neodiprion sertifer nucleopolyhedrovirus. J Virol 78: 7036-7051.

73. Afonso CL, Tulman ER, Lu Z, Balinsky CA, Moser BA, et al. (2001) Genome sequence of a baculovirus pathogenic for Culex nigripalpus. J Virol 75: 11157-11165.
